# Assessment of porcine reproductive and respiratory syndrome virus (PRRSV) farm surface contamination through environmental sampling

**DOI:** 10.1186/s40813-024-00387-5

**Published:** 2024-09-27

**Authors:** Claudio Marcello Melini, Mariana Kikuti, Laura Bruner, Matt Allerson, Katie O’Brien, Chase Stahl, Brian Roggow, Paul Yeske, Brad Leuwerke, Mark Schwartz, Montserrat Torremorell, Cesar A. Corzo

**Affiliations:** 1 Department of Veterinary Population Medicine, University of Minnesota, St. Paul, MN USA; 2 Swine Vet Center, St. Peter, MN USA; 3 Holden Farms Inc, Northfield, MN USA; 4 Fairmont Vet Clinic, Fairmont, MN USA; 5 Schwartz Farms, Sleepy Eye, MN USA

**Keywords:** PRRS, Environmental sampling, Surfaces, Contamination

## Abstract

**Background:**

During the fall of 2020, the porcine reproductive and respiratory syndrome virus (PRRSV) L1C.5 variant emerged and rapidly spread throughout southern Minnesota generating questions regarding possible transmission routes. This study aimed to investigate whether PRRSV could be detected on surfaces inside and outside pig barns housing L1C.5 variant PRRSV-positive pigs to illustrate the potential for indirect transmission of PRRSV. Seven Midwestern U.S. PPRS-positive breeding or growing pig farms and one PRRS-negative farm were conveniently selected. Internal and external barn surfaces were wiped using a PBS moistened cloth and the resulting liquid was submitted to the University of Minnesota Veterinary Diagnostic Laboratory for PRRSV RT-PCR testing and virus isolation.

**Results:**

All (*n* = 26) samples from PRRSV-negative farm tested negative. Nineteen (13%) out of 143 samples from positive farms yielded positive RT-PCR results. Positive samples originated primarily from exhaust fan cones and doorknobs, followed by anteroom floor and mortality carts/sleds. Virus isolation attempted on two samples did not yield positive results.

**Conclusions:**

PRRSV contamination can occur on surfaces inside and outside pig barns that are in frequent contact with farm personnel. Although virus isolation attempts were negative, our results illustrate the potential for PRRSV to be transmitted indirectly through contaminated materials or farm personnel. The study supports the implementation of biosecurity practices by farm personnel to prevent the introduction of PRRSV into farms and the prevention of PRRSV transmission between farms.

## Background

Porcine reproductive and respiratory syndrome (PRRS) is an endemic and costly disease affecting swine in the United States (U.S.) and throughout the world [1, 2]. The causative agent of the disease is a 15 kb single-stranded positive-sense RNA virus belonging to the *Arteriviridae* family and *Arterivirus* genus [3, 4]. The PRRS virus (PRRSV) can be classified into two species, *Betaarterivirus suid 1* (PRRSV-1) or *Betaarterivirus suid 2* (PRRSV-2) [5]. In the breeding herd, PRRS is characterized by reproductive failure, increased abortions, stillbirths, mummies, premature farrowing, and weak-born piglets. PRRSV also causes respiratory disease characterized by interstitial pneumonia which may result in mortality and poor growth performance [6].

PRRSV can be directly transmitted between infected and susceptible animals through saliva, nasal and oral secretions, feces, urine, semen, and mammary gland secretions. Secretions containing infectious PRRSV can also contaminate inanimate objects which contribute to the indirect transmission of PRRSV [7, 8]. However, the ability of PRRSV to transmit indirectly depends on environmental conditions such as temperature (-10 to 20 °C), pH (6.5 to 7.5), and humidity (17 to 73%), virus variant, type of contaminated surface, and exposure to chemicals (detergents and lipid solvents) [3, 4, 6, 9–18]. Indirect transmission in pigs may occur as a consequence of exposure to contaminated fomites such as boots, coveralls, needles, and transport vehicles [10, 19–25], and aerosols [20, 23, 26–28].

In the U.S., PRRS occurs seasonally with increased incidence typically beginning in October-November and receding into spring [29, 30]. During the fall of 2020, a new PRRSV variant classified as sub-lineage 1C (L1C.5) emerged in southern Minnesota and multiple pig farms from various pig production companies were affected. Approximately 6 months later in the spring-summer of 2021, a second wave of PRRSV outbreaks was reported [31] which was atypical for the U.S. swine herd. Furthermore, farms with robust biosecurity measures (e.g., air filtration, shower in-shower out, disinfection and drying [D&D] room, visitor downtime, Danish entry system) and located in areas considered of low infection risk (i.e., low pig density) became positive with this variant. Because of the rapid transmission of this PRRSV variant throughout a wide geographic area and the absence of specific risk factors associated with this variant’s occurrence [31], it is hypothesized that farms were becoming infected through indirect routes breaching biosecurity measures.

Because there is limited information on what surfaces may be more likely to be contaminated with PRRSV in swine farms, we sampled surfaces considered of high risk of contamination inside and outside farms housing PRRS-L1C.5 positive pigs to better understand the potential risk of PRRSV dissemination from infected premises.

## Methods

### Study design

This cross-sectional study was conducted during the summer-fall of 2021 and spring of 2022 in which seven farms were conveniently selected. Farm eligibility criteria included: (1) breeding or growing pig farms representative of modern pig production practices in the U.S., (2) laboratory confirmation by RT-PCR and ORF5 sequencing that pigs were infected with PRRSV L1C.5 variant, (3) pigs were in the early stages of the outbreak (i.e., 4 to 5 weeks post-estimated virus introduction), and (4) farms located in the Midwestern U.S. In addition, one PRRSV negative (i.e., PRRSV naïve, AASV Breeding herd classification status 4) farm located in the epicenter of the outbreak and in a high pig-dense area was identified and included in the study as a negative control.

### Sample size and surfaces

The study was designed to assess whether PRRSV RNA could be detected on different surfaces of pig farms through RT-PCR. Given that, there were no available protocols for assessing viral contamination in pig farms, a sampling methodology was developed in conjunction with practicing veterinarians. A maximum of 29 samples per farm were collected. This sample size allowed a 95% confidence level in detecting at least one positive sample when the prevalence of positive surfaces was estimated to be at least 10%. Surfaces included for sampling were not in direct contact with pigs and were chosen based on the likelihood of contamination (Table 1), materials present at the farm (e.g., rubber, concrete, plastic, wood, metal cloth) [10, 32–35] and risk that these surfaces could then be in contact with farm personnel.

**Table 1:** Sampling approach to detect PRRSV on surfaces from United States Midwestern pig farms housing PRRSV-positive pigs

Sampling areas		Number of Samples	
		Breeding farm	Growing/finishing farm
Employee vehicles			
	Exterior door handles	6 to 12*	2 to 4*
	Pedals and footrest	9	3
	Car exterior surfaces (roof, trunk, door facing the farm)	3	3
Main Entrance doorknobs			
	Exterior and interior doorknobs of the farm’s main entrance door	1	1
	Both doorknobs of the door used to bring mortality out of the barn	1	1
Anteroom			
	Floor directly in front of the main door	1	1
	Floor closest to the bench or shower door	1	1
D&D Room			
	Floor inside the D&D room area directly in front of the exterior door Both doorknobs of the exterior door of the room	1 1	1 1
Exhaust fan housing			
	Fan housing and fan louvers/shutters	3	2
Loading chute			
	Edge (that comes in contact with the trailer) of the loading ramp.	1	1
Particle deposition			
	Aluminum foil in 4 locations around the farm (North, South, West, and East) 30 m away from the barn.	4	4
Other			
	Mortality sled or cart	1	1

*Quantity depended on the number of vehicles present during the sampling day

### Environmental sample collection

Investigators wore disposable protective suits (Tyvek^®^, Dupont™, Wilmington, DE, USA) and plastic boot covers upon arrival at the farm site and before getting out of the vehicle. Hands were cleaned with disinfectant wipes before sampling begun and between samples. A clean set of nitrile rubber gloves was worn before collecting each sample. Samples were collected using a previously validated environmental sampling protocol for PRRSV [36, 37]. Briefly, under clean laboratory conditions a sterile gauze or dry cloth (Swiffer^®^, P&G, Cincinnati, OH, USA) was placed inside a new resealable plastic bag (Ziploc^®^, S.C. Johnson, Racine, WI, USA) and moistened with 20 mL of phosphate-buffered saline (PBS) solution. Using new disposable nitrile rubber gloves, the cloth was removed from the resealable bag at the farm, the surface was wiped and the cloth was placed back into the original bag and sealed. Samples were collected using a 2-step approach. First, four locations around the pig barn representing the cardinal points were chosen. At each of these locations, a sheet of aluminum foil paper (1 m x 0.3 m) was placed on the ground 30 m away from the barn. The aluminum foil paper was then wiped with the sampling gauze 60 min after placement. Second, outside the barns and in the anteroom flooring, surface samples were then collected by wiping a 0.30 m x 0.30 m area of the surface (e.g., floor) or the whole surface (e.g., doorknobs, door handles, car pedals). The bag, together with the cloth, were squeezed and the excess fluid was poured into a sterile plastic falcon tube (Corning Falcon^®^, ThermoFisher Scientific Inc., Waltham, MA, USA). Samples were labeled with a specific identifier according to the surface wiped, farm identification number and placed into a refrigerated container.

### Sample testing

Samples were transported to the University of Minnesota Veterinary Diagnostic Laboratory (UMN-VDL) for processing and testing. Samples were vortexed (Analog Vortexer Mixer, ThermoFisher Scientific Inc., Waltham, MA, USA) for 15 to 20 s at 3200 rpm before the extraction process. High throughput total nucleic acid extraction was done using magnetic bead technology (MagMAX™ CORE Nucleic Acid Purification Kit, ThermoFisher Scientific Inc., Waltham, MA, USA), and PRRSV RT-PCR was performed using VetMAX™ PRRSV EU & NA v3.0 kit following the manufacturer’s instructions. Based on UMN-VDL protocols, samples below an RT-PCR cycle threshold (Ct) value of 40 were considered positive. Virus isolation (VI) was attempted on MARC-145 and porcine alveolar macrophages (PAM) on samples yielding a Ct value below 30 using a previously described protocol [38].

## Results

A total of 169 environmental samples were collected from all 8 farms, 75 from three breeding herds, and 94 from growing pig farms (i.e., nursery or wean-to-market farms). The number of samples collected at each farm was affected by the presence or absence of specific sampling surfaces (e.g., two-door vehicles instead of four-door, absence of D&D room, absence of vehicles during visit).

From the negative control breeding farm, all 26 samples yielded RT-PCR negative results. In six out of the seven farms housing PRRSV-positive pigs, at least one surface yielded an RT-PCR-positive result. Of the 143 samples obtained from positive farms, 19 (13.2%) samples yielded RT-PCR positive results with Ct values ranging between 25.4 and 37.0 (Table 2). Of the 19 positive samples, 15 (79%) originated from non-porous surfaces such as plastic or metal whereas 4 (21%) from porous materials such as concrete. Eight (42%) out of the 19 positive samples originated from exhaust fan cones in four farms and had a Ct value ranging from 30.3 to 37.0. The main door entry doorknobs or the doorknobs of the door leading to the mortality disposal corridor at three farms tested RT-PCR positive with Ct values ranging between 34.2 and 36.4. The remaining eight positive samples originated from surfaces from the anteroom floor and mortality carts/sleds with Ct values ranging from 25.4 to 35.4. None of the foil paper particle deposition samples tested positive for PRRSV.

The two samples originating from mortality sleds that had a Ct value below 30 were further tested by virus isolation on MARC-145 and PAM cells yielded negative results.

**Table 2:** Farm characteristics and environmental sample PRRSV RT-PCR results originating from seven L1C.5 positive farms and one negative farm

Farm ID	Farm type	Herd size	Positive samples/total samples (%)	Location of each positive sample	PRRSV RT-PCR Cycle threshold (Ct) values
1*	Breeding*	1500	0/26 (0%)	--	--
2	Breeding	900	2/26 (8%)	Exhaust fan cone	30.3
				Exhaust fan cone	37.0
3	Nursery/Finisher	2400–4800	1/23 (4%)	Exhaust fan cone	35.9
4	Breeding	2678	0/23 (0%)	--	--
5	Nursery	6600	5/23 (22%)	Exhaust fan cone	34.0
				Exhaust fan cone	34.4
				Exhaust fan cone	36.0
				Anteroom floor next to the bench’s dirty side	35.4
				Mortality doorknob	36.4
6	Nursery	2400	1/15 (7%)	Main entrance doorknob	34.2
7	Wean-to-market	2400	2/18 (11%)	Mortality loading chute internal floor	31.7
				Mortality sled**	25.4
8	Wean-to-market	2400	8/15 (53%)	Exhaust fan cone	32.3
				Exhaust fan cone	34.8
				Mortality loading chute internal doorknob	35.0
				Anteroom floor closest to the side of the line of separation to pens	33.6
				Mortality sled #1**	28.7
				Mortality sled #2 handle	33.3
				Mortality sled #2	33.7
				Outside barn entrance floor	31.5

*Negative control farm, **Samples submitted for virus isolation

## Discussion

Our study confirmed that detecting PRRSV RNA from surfaces in farms undergoing a PRRSV L1C.5 variant outbreak was possible. The surfaces were not in direct contact with pigs and represented surfaces that employees frequently come in contact with (e.g., doorknobs and anteroom flooring). Our results highlight the importance of barn entry and exit processes directed at limiting the contact of personnel with contaminated surfaces.

Even though efforts were made to assess whether the virus was viable by virus isolation, none of the two tested samples yielded positive results. Viability data of swine viruses on surfaces exists for influenza A virus (IAV) and porcine epidemic diarrhea virus (PEDV) concluding that these can remain viable longer on nonporous surfaces when compared to porous surfaces [39, 40]. Failure to detect viable virus could be the result of damaged RNA due to factors such as UV-light exposure, desiccation, time, humidity, and temperature [3, 4, 6, 9, 11, 41]. In addition, it was not known for how long these viral particles had been present on these surfaces prior to sample collection, which ultimately could have limited the probability of detecting virus.

Almost half (42%) of the RT-PCR positive results originated from exhaust fan cones which indicated that virus particles originating from animals became airborne and were expelled from the barn through the exhaust fans and some were deposited on the cone surface. This information suggests that biocontainment of PRRSV can be complex and environmental contamination can occur in the immediate vicinity of the affected farms. Even though our study detected viral RNA on exhaust fan cone surfaces, our particle deposition sampling did not yield positive results. This conflicting result could be due to a dilution effect, or dispersion over greater distances because of wind gusts during sampling or limited sampling time. Furthermore, viral particles on exhaust fan cone surfaces had accumulated over a longer period of time and had higher proximity infected pigs when compared to our aluminum foil particle deposition approach which ultimately may have led to the virus being detected. This accumulation may also be the result of deficient or lack of cleaning protocols including these surfaces.

Detecting PRRSV RNA on surfaces that farm personnel contact frequently was revealing. Surfaces such as the anteroom floor or doorknobs had positive RT-PCR results which can be the result of farm personnel likely contaminating these surfaces through footwear, dirty gloves, or hands. The detection of viral RNA on the floor closest to the bench in the anteroom is concerning from a biocontainment perspective. This finding could be the result of employees either bringing the virus into the farm through contaminated footwear or contaminated footwear being moved across the bench from the clean side towards the dirty side of the bench. However, given our study design the likelihood of the detected RNA originating from the inside is higher, but it cannot be determined. Another possibility is that airborne virus could have settled on these surfaces. In the case of doorknobs, employees can easily contaminate their hands or gloves during pig or equipment handling and subsequently transfer the virus to doorknobs. Otake et al., [25] and Pitkin et al., [21] were able to detect PRRSV by RT-PCR in samples obtained from coveralls, boots, hands, feed bags, cable snare, and blood-testing supplies; however, viable virus was not recovered. Therefore, our results agree with previous reports and continue to highlight the fact that the virus can potentially be transported by farm personnel out of the barns which becomes a risk for PRRSV dissemination.

Findings from this study underscore the importance of biocontainment during PRRSV outbreaks. Detecting viral RNA on samples outside the barn (e.g., exhaust fan cones, mortality sled, anteroom floor, outside the barn floor) indicates that the virus is potentially being carried out of the barn which may ultimately lead to increased risk to neighboring farms. The mortality sled being contaminated with PRRSV during an outbreak or weeks after may be expected because its surfaces come in direct contact with blood, secretions, and excretions containing high viral loads. These surfaces may be considered a source of virus but also highlight the need for a better understanding of the risks related to mortality management, especially processes related to dead animal removal from the barn and dead pig transport to the mortality pick-up location as it has been previously reported [42, 43]. Additional data is needed to assess the presence and PRRSV viability on the surface of the mortality sled and mortality pick-up locations under different environmental conditions.

As with any research study, our study has limitations. Our sampling protocol was designed using current environmental sampling knowledge and likely our sample size may have led us to underestimate the detection of the virus on surfaces. In addition, the protocol was calculated to originally collect a minimum of 209 samples, and only 169 samples were collected, mostly due to the lack of presence of the targeted surfaces, which can affect the power of the study. Another limitation is the fact that even though we knew the population of pigs housed in these barns was undergoing an outbreak we did not know the extent of viral shedding by the animals at the time of collection which can play a role in environmental contamination.

## Conclusions

The detection of PRRSV genetic material on surfaces on the inside and outside surfaces of a farm housing PRRSV-positive pigs is possible using targeted surface sampling. This exploratory study provides evidence that biocontainment efforts to prevent the spread of PRRSV from infected farms are necessary.

## Data Availability

No datasets were generated or analysed during the current study.
